# Analysis of Deep Vein Thrombosis: A Prospective Observational Study

**DOI:** 10.7759/cureus.68014

**Published:** 2024-08-28

**Authors:** Iqbal M Ali, Vijay Sai Reddy M, Varun Shetty

**Affiliations:** 1 General Surgery, Dr D. Y. Patil Medical College, Hospital and Research Centre, Dr. D. Y. Patil Vidyapeeth, Pune, IND

**Keywords:** deep vein thrombosis (dvt), thrombolytic therapy, anticoagulation, stasis, dvt, hypercoagulability, thrombosis, thrombosis

## Abstract

Objective

This prospective observational study aimed to investigate the prevalence, acquired risk factors, and treatment outcomes of deep vein thrombosis (DVT) at a tertiary care center in India.

Materials and methods

Conducted from 2022 to 2024, the study included 100 patients diagnosed with lower extremity DVT. The study subjects included patients visiting the general surgery, vascular surgery, surgical gastroenterology, general medicine, emergency medicine, and obstetrics and gynecology departments with symptoms suggestive of DVT. The primary objectives were to evaluate the effectiveness of conventional anticoagulation and thrombolytic therapy as well as to assess the various acquired risk factors for DVT. Patients underwent comprehensive clinical and biochemical evaluations, including venous Doppler ultrasound, and were treated based on their clinical presentations. The study's primary end outcome was early recanalization rates on day 14 post-initiation of treatment and occurrence of post-thrombotic syndrome (PTS). Secondary outcome measures included duration of hospital stay, time taken to return to work and early complications.

Results

The highest incidence of DVT was in individuals in their forties, with a mean age of 44.84+/-11.71 years and a female preponderance of 58% (n=58). Key acquired risk factors identified included hypertension (25%; n=25), diabetes mellitus (20%; n=20), obesity (16%; n=16), and smoking (34%; n=34). Obesity (16%; n=16), a history of DVT (25%; n=25), trauma/immobilization (9%; n=9), pregnancy (10%; n=10), smoking (34%; n=34), and cancer (20%; n=20) were also identified as important acquired risk factors contributing to the occurrence of DVT. Amongst the study participants, 28% (n=28) had femoro-popliteal segment involvement, 36% (n=36) had calf vein thrombosis, and the remaining 36% (n=36) showed femoro-iliac segment thrombosis. Conventional anticoagulation was administered to 69% (n=69) of patients, while 31% (n=31) received thrombolytic therapy. Both treatments showed similar recanalization rates, but thrombolytic therapy was associated with a longer hospital stay (8.61+/-1.65 days; p=0.024; p<0.05) and return to work period (14.65+/-2.31 days; p=0.012; p<0.05). Post-thrombotic syndrome was less common in the thrombolytic therapy group (3%; n=3). Three patients died in the study, with the cause being pulmonary embolism. The descriptive statistics were computed to delineate the study sample. After completion of data collection, data analysis was achieved using SPSS for Windows, Version 16 (Released 2007; SPSS Inc., Chicago, United States), and the correlations sought after were achieved using the chi-square test of significance.

Conclusion

The study underscores the importance of recognizing and managing acquired risk factors for DVT, thereby facilitating early diagnosis and ultimately reducing morbidity and mortality. Understanding these factors and employing effective treatment strategies are crucial for better management and prevention of DVT, enhancing patient outcomes.

## Introduction

Venous thromboembolism (VTE), which includes deep vein thrombosis (DVT) and pulmonary embolism (PE), ranks as the third most prevalent and dangerous cardiovascular disease [1]. The worldwide annual occurrence of DVT is approximated to be one to two cases per 1000 individuals. The yearly occurrence of DVT in India has been estimated to be one percent of the adult population aged forty and above and 15-20% among patients who are admitted to hospitals [2]. The lack of an accurate diagnosis of DVT or PE poses a significant risk of morbidity and mortality, both in the short as well as long-term. Misdiagnosis of DVT can also lead to the progression of DVT, the occurrence of new PE, and long-term financial strain.

The morbidity linked to DVT is primarily due to the development of post-thrombotic syndrome (PTS). This condition impacts a substantial proportion of patients, with as many as 50% encountering symptoms such as leg pain, swelling, and, in severe instances, venous ulcers within two years of being diagnosed with DVT [3]. While our comprehension of the pathophysiology of DVT has made significant progress in recent years, there are still crucial concerns regarding the application of this knowledge to improve clinical outcomes [4-6]. The main objective of anticoagulation therapy for DVT is to prevent the development of PE and the recurrence of blood clot formation (thrombosis). The mortality rate within 30 days for individuals with DVT who are not receiving anticoagulation treatment is more than 3%. This risk becomes ten times higher for those who also develop PE [7]. The advent of direct oral anticoagulants (DOACs) has prompted a need to compare these newer drugs with the more conventional vitamin K antagonists (VKAs) for the management of DVT. DVT is a complex condition that is influenced by a combination of genetic and environmental risk factors. With the increasing prevalence of these well-known risk factors in society, VTE has emerged as a significant public health concern [8,9]. Therefore, it is crucial to promptly and accurately diagnose DVT and diligently apply appropriate treatment methods in order to prevent the associated morbidity and the development of complications. The aim of this study was to identify and discuss the different acquired risk factors that make individuals more likely to develop DVT. The study also aimed to compare the treatment outcomes of conventional anticoagulation and thrombolytic therapy (including catheter-guided thrombolysis), which are two commonly employed modalities to combat DVT.

## Materials and methods

We conducted this prospective observational research at Dr D. Y. Patil Medical College, Hospital and Research Centre, Dr. D. Y. Patil Vidyapeeth, Pune, a tertiary care health center between 2022 and 2024. The investigation included 100 consenting study participants presenting to our center with DVT involving the lower extremities. The study subjects included patients visiting or admitted in general surgery, vascular surgery, surgical gastroenterology, surgical oncology, general medicine, emergency medicine, and obstetrics and gynecology departments of our institute with symptoms suggestive of DVT. We obtained institutional ethics clearance (I.E.S.C./328/2022) prior to beginning the study. The study was initiated only after obtaining informed consent from all the participants. 

The diagnostic criteria employed for inclusion in the study were based on the presence of clinical/ultrasound (Doppler) features indicative of DVT (including the absence of flow on spectral color Doppler, static valve leaflets, and absent compressibility of the vein). The study excluded patients with chronic DVT and hereditary coagulopathies prior to inclusion in the study since the primary focus of the study was on acquired risk factors. We thoroughly evaluated all the included participants through a detailed clinical examination and biochemical evaluation, which included a complete blood count, serum electrolytes, renal and liver function tests, and coagulation profile. We recorded these findings in a dedicated pro forma created for this research. On admission, the patients underwent a primary venous color Doppler to ascertain the presence and level of DVT. The level of DVT was divided into the involvement of the inferior vena cava, femoro-iliac segment, femoro-popliteal segment, and lastly, calf thrombosis. Patients with more proximal DVT were considered more severe, with a higher risk for pulmonary embolism.

The study documented the demographic profile and the contributory acquired risk factors for DVT. These acquired risk factors included age, gender, occupation, body mass index (BMI), co-morbidities (diabetes mellitus (DM), hypertension (HTN), chronic obstructive pulmonary disease (COPD), cancer, previous history of DVT, surgical history (major or minor), trauma and immobilization, pregnancy, smoking, and drug history (estrogen-containing oral contraceptives, chemotherapeutic agents). Hereditary risk factors, including coagulopathies such as thrombophilia, also constitute important risk factors for DVT but were beyond the purview of this study. Depending on the surgeon's preference and the severity of the DVT, we treated all patients with acute DVT with either conventional anticoagulation (unfractionated heparin (UFH), low molecular weight heparin (LMWH), or thrombolytic therapy (including catheter-guided thrombolysis).

Conventional anticoagulation included administering UFH or LMWH. The patient received a bolus of 5000 units of UFH followed by a continuous infusion, titrated according to activated partial thromboplastin time (aPTT). After five days, the patients were maintained on oral anticoagulants. Thrombolytic therapy included parenteral as well as catheter-guided thrombolysis with agents such as alteplase/reteplase. These treatment modalities were then compared, with the endpoints of comparison being early recanalization, duration of hospital stay, time to get back to work, and the development of complications (including pulmonary embolism and death). The patients after discharge from the hospital were followed up on days seven, 10, and 14 to observe the rate of early recanalization.

We computed the descriptive statistics to define the study sample. Once the data collection was complete, we used the SPSS for Windows, Version 16 (Released 2007; SPSS Inc., Chicago, United States) to analyze the data and achieve the desired correlations using the independent ‘t’ test of significance.

## Results

The fourth decade had the highest incidence of DVT in our study, accounting for 41% (n=41). The study participants' average age was 44.84 +/- 11.71 years (mean +/- standard deviation), as shown in Table 1. Of the 100 patients, 58% (n=58) were women and 42% (n=42) were men. The observed sex ratio was 72.41 males to 100 females, indicating a slight female preponderance. The majority of participants (40%; n=40) worked in a semi-skilled occupation, with housewives accounting for the second largest occupational group (34%; n=34). According to the modified Kuppuswami scale, the majority of study participants were from the lower middle (30%; n=30) and lower (25%; n=25) socioeconomic strata.

**Table 1:** Age-wise distribution of study participants (in years)

Age groups	Frequency	Percentage
21-30	15	15
31-40	16	16
41-50	41	41
51-60	16	16
> 61	12	12
TOTAL	100	100

The study participants were subjected to an initial Doppler assessment, based on which they were divided into cases with DVT involving the femoro-iliac segment (36%; n=36), femoro-popliteal segment (28%; n=28), or limited calf vein thrombosis (36%; n=36). The cases with proximal DVT (36%; n=36) were considered more severe based on a higher propensity for pulmonary embolism. However, no objective scoring system was employed to assess the severity of DVT. The majority of our study participants, 38% (n=38), had no comorbidities, but 25% (n=25) had HTN, 20% (n=20) had DM, and 17% had hypertriglyceridemia, all of which are considered risk factors for DVT. Obesity (16%; n=16), a history of DVT (25%; n=25), trauma/immobilization (9%; n=9), pregnancy (10%; n=10), smoking (34%; n=34), and cancer (20%; n=20) were also identified as acquired risk factors. Around 30% of the study subjects had undergone a major/minor surgical procedure prior to the development of DVT, with pelvic surgeries accounting for the majority (63%; n=18), followed by non-pelvic surgeries (37%; n=12). Furthermore, the use of medications such as estrogen-containing oral contraceptives (20%; n=10) and chemotherapeutic agents (6%; n=6) was identified as a contributory, acquired risk factor for DVT.

Among the 100 patients, 69% (n=69) received conventional anticoagulation, and 31% (n=31) received thrombolytic therapy (including catheter-guided thrombolysis). Thrombolytic therapy resulted in complete or partial recanalization in 87.1% of patients (n=27), while 12.9% (n=4) did not recanalize and required additional treatment. On the other hand, conventional anticoagulation resulted in complete or partial recanalization in 94.2% (n=65) of participants, while 5.8% (n=4) required additional treatment. Fisher's exact test revealed a p-value of 0.408, indicating that the relationship between the type of intervention and 14th-day imaging results (in terms of recanalization) was not statistically significant (Table 2). Thus, anticoagulation and thrombolytic/endovascular therapies produced comparable follow-up imaging results.

**Table 2:** Association of different treatment modalities of DVT with recanalization rate on day 14 post-therapy End point compared: recanalization post-administration of treatment. The Fisher's exact test was utilized to establish a correlation between the type of intervention and the rate of recanalization. The p-value obtained was 0.401 for the conventional anticoagulation group. Similarly, the p-value for the thrombolysis group was 0.408, respectively. However, considering p<0.05 as the end point for statistical significance, both modalities had a comparable recanalization rate on day 14. DVT: deep vein thrombosis

Categories	Complete/Partial recanalization	No change	Total	Fisher exact	P-value
	N (%)	N (%)	N (%)	0.204	0.401 +/- 0.004 (conventional anticoagulation group), 0.408 +/- 0.002 (thrombolysis group)
Thrombolytic therapy including catheter guided thrombolysis (endovascular treatment)	27 (87.1)	4 (12.9)	31 (31)		
Conventional anticoagulation	65 (94.2)	4 (5.8)	69 (69)		
Total	92 (92)	8 (8)	100 (100)		

In our study, patients treated with conventional anticoagulation had an average hospital stay of 7.52 ± 2.94 days, while those treated with endovascular/thrombolytic therapy had an average of 8.61 +/- 1.65 days. The student's t test found a significant correlation between the type of intervention and hospital stay duration (p=0.0244; p<0.05). Patients who received endovascular thrombolytic therapy had a significantly longer time to return to work (14.65 ± 2.31 days) than those who received conventional anticoagulation (13.06 ± 3.74 days), as shown in Table 3. The difference was statistically significant (p=0.012). Only two patients (6.45%) developed PTS after receiving thrombolytic therapy, compared to nine patients (13.04%) who received conventional anticoagulation. Only 3% of the study patients developed PE, and all three died.

**Table 3:** Association of different treatment modalities of DVT with duration of hospital stay and time taken to get back to work End points for comparison: duration of hospital stay (in days) and time taken to get back to work (in days). The statistical analysis included an independent 't' test to assess the association between the two variables. The p-value for the duration of hospital stay was 0.024, which was less than the level of significance of 0.05. This indicates that there was a statistically significant difference in the duration of hospital stay among both types of procedures. Similarly, the p-value for the time taken to return to work was 0.012 (p<0.05), indicating a statistically significant association. DVT: deep vein thrombosis

	Conventional anticoagulation	Thrombolytic therapy including catheter guided thrombolysis (endovascular treatment)	T-value	P-value
	MEAN ±SD	MEAN ±SD	2.28	0.0244
Duration of hospital stay	7.52±2.94	8.61±1.65		
Time to get back to work	13.06±3.74	14.65±2.31	2.55	0.012

## Discussion

DVT is one pole of the VTE continuum, with PE representing the other. It presents a clinical challenge for doctors from all disciplines. It can exacerbate an illness's progression, but it can also occur in the absence of triggering conditions. Thrombosis can occur anywhere in the venous system, but it is most common in the deep veins of the lower extremities. The main concern, however, is the embolization of the thrombus into the lung, which can be fatal [10].

DVT is a major public health concern in India, resulting in high preventable morbidity and mortality rates, as well as a substantial financial burden. A number of factors acting alone or in tandem are responsible for the higher rates of DVT in Western countries compared to India and other Asian countries [11]. These include a genetic predisposition to thrombophilia, lifestyle factors such as low physical activity and increased obesity rates, an aging population, and environmental factors such as climate and seasonal variations.

Our study's mean age was 41.84 +/- 11.71 years, which is consistent with the findings of Kakkar N et al. and Samama et al. [12,13]. This is due to the prevalence of underlying causes in this age group, such as cancer, reduced physical activity, and prothrombotic conditions. In our study, 58% of the patients were women (n=58). Previous studies, including those conducted by Lee et al. and Baglin et al., had a higher proportion of female participants (Table 4) [14,15]. This phenomenon may be caused by obesity, gravid uterus, hypercholesterolemia, and hormonal changes in females, which may be the root causes of this phenomenon. Increased use of oral contraceptives and hormonal therapy, as well as high pregnancy rates among Indian women, could all contribute.

**Table 4:** Age and gender distribution in studies conducted on DVT DVT: deep vein thrombosis

Name of study	Mean age (in years) and Gender (Male : Female)
Kakkar et al. [12]	40.28 +/- 10.20 years with male preponderance (1.5:1)
Samama et al. [13]	44.24 +/- 11.22 years with female preponderance (1:1.2)
Lee et al. [14]	50.23 +/- 10.12 years with female preponderance (1:1.5)
Baglin et al. [15]	40.21 +/- 12.10 years with female preponderance (1:1.4)

HTN (25%) was the most common co-morbidity in this patient group, followed by DM (20%), obesity (16%), and hypertriglyceridemia (17%), which was consistent with the findings of Dhanesh K et al. [16]. Prior history of DVT was the second most common risk factor (34%) in patients with only DVT. Those with a history of VTE are about eight times more likely to have a new episode during a future high-risk period than those with no history of DVT or PE [17].

The next acquired risk factor assessed was prior history of surgery. Among the study subjects with DVT included in our research, 30% (n=30) had undergone major/minor surgery, following which they developed DVT. Out of these, 63% (n=18) of patients underwent pelvic surgery (including obstetric/gynecological and cancer procedures), while 37% (n=12) had non-pelvic surgeries. Our findings appear to be consistent with those reported by Dhanesh K et al. in his registry, who identified major lower limb surgery followed by pelvic surgery as a significant risk factor for DVT [16]. Furthermore, he discovered that laparoscopic surgery resulted in a significant reduction in DVT occurrences compared to open surgery. Other factors associated with varying risk for DVT are patient placement and immobility following surgery or trauma [18]. Out of the 100 included patients with DVT, 20% (n=20) were admitted to the gynecology and surgical oncology departments with systemic malignancies, of which genitourinary and gynecological cancers were the most common (52%). The most common presenting symptom of these patients was lower limb cramping pain in addition to the symptoms associated with their primary pathologies. Smoking poses a significant risk of DVT. Our investigation found that 28% of the patients smoked, which is consistent with the findings of Kakkar et al. [12]. Smoking, while not a direct risk factor, increases the patient's risk of DVT through hypercoagulability and atherosclerosis.

Amongst our study participants, 80% had a past history of COVID-19, and 98% of these had received two doses of the vaccine. Dhanesh K et al. demonstrated that post-discharge DVT risk decreased from 3% in the pre-Delta wave to 1.7% in the Delta and 0.9% in the Omicron wave [16]. The widespread use of anticoagulants and vaccinations could explain the decrease in VTE risk. In our study, 69% of patients received traditional anticoagulants. Around 31% of patients received thrombolytic treatment, which included catheter-directed thrombolysis. The most recent VTE/DVT treatment guidelines state that patients with acute deep vein thrombosis in their lower extremities are more likely to benefit from thrombolytic therapy [19].

In our study, only two out of 31 patients who received thrombolytic therapy developed PTS. Of the 69 patients who received medical attention, nine developed PTS while on anticoagulant therapy. Only 3% (n=3) of the patients died, with the common cause being PE. These findings were consistent with those reported by Bergquist et al. [20].

In our study, thrombolytic/endovascular treatment was significantly associated with higher recanalization rates than traditional anticoagulation. Steven et al. conducted similar research but found no significant relationship (p > 0.716) [21]. Anticoagulation and thrombolytic therapy, like any other medical treatment, have their own set of drawbacks, the most serious being excessive bleeding. Magdalena et al. discovered that using thrombolytics increased the risk of severe bleeding and pulmonary embolism within 24 hours. However, our investigation revealed no bleeding incidents [22].

Our research employed appropriate inclusion/exclusion criteria as well as statistical methods in order to identify important acquired risk factors for DVT. We have managed to include a wide spectrum of patients, presenting to not only general surgery but other departments such as vascular surgery, surgical gastroenterology, surgical oncology, general medicine, emergency medicine, and obstetrics and gynecology departments. A more detailed study with a longer duration of follow-up as well as more confounding variables is planned in the near future.

The study has several flaws, including a small sample size, a lack of bias prevention techniques, and a brief follow-up period. Also, hereditary risk factors such as thrombophilia were not included in the study. Nonetheless, we intend to broaden our research by enrolling a larger study cohort, resulting in more comprehensive data that will add to the growing body of knowledge about acute DVT and its management.

## Conclusions

DVT represents a major preventable cause of morbidity and mortality. Our research demonstrated prior history of VTE, obesity, comorbidities (including DM/HTN/COPD), and cancer as predominant acquired risk factors for DVT. In addition, it also highlighted pelvic surgeries, trauma/immobilization, and oral contraceptives as significant risk factors contributing to the occurrence of DVT. Hereditary risk factors, including thrombophilia, also significantly contribute to the occurrence of DVT. However, these hereditary risk factors were beyond the purview of this study. Taking notice of these acquired risk factors can help not only in the early detection of DVT but also can guide surgeons to be more vigilant in taking measures to prevent DVT. Both conventional anticoagulation as well as thrombolytic therapy were comparable in outcomes; however, thrombolytic therapy was associated with a lower incidence of early-onset PTS/hemorrhage. However, a longer duration of follow-up is needed for a more categorical demonstration of the benefit of either treatment modality in this context. Furthermore, a thorough understanding of the benefits and drawbacks of conventional anticoagulation/thrombolysis in DVT treatment can assist the surgeon or physician in making a more informed decision, lowering the impact of DVT on morbidity and mortality rates. 
